# Cellular Reparative Mechanisms of Mesenchymal Stem Cells for Retinal Diseases

**DOI:** 10.3390/ijms18081406

**Published:** 2017-07-28

**Authors:** Suet Lee Shirley Ding, Suresh Kumar, Pooi Ling Mok

**Affiliations:** 1 Department of Biomedical Science, Faculty of Medicine and Health Sciences, University Putra Malaysia, 43400 UPM Serdang, Selangor, Malaysia; suetlee.ding@gmail.com; 2 Department of Medical Microbiology and Parasitology, Faculty of Medicine and Health Sciences, Universiti Putra Malaysia, 43400 UPM Serdang, Selangor, Malaysia; sureshkudsc@gmail.com; 3 Genetics and Regenerative Medicine Research Centre, Universiti Putra Malaysia, 43400 UPM Serdang, Selangor, Malaysia

**Keywords:** mesenchymal stem cells, retinal degenerative diseases, MSC differentiation, paracrine activity, anti-inflammatory, immunomodulatory, anti-angiogenesis

## Abstract

The use of multipotent mesenchymal stem cells (MSCs) has been reported as promising for the treatment of numerous degenerative disorders including the eye. In retinal degenerative diseases, MSCs exhibit the potential to regenerate into retinal neurons and retinal pigmented epithelial cells in both in vitro and in vivo studies. Delivery of MSCs was found to improve retinal morphology and function and delay retinal degeneration. In this review, we revisit the therapeutic role of MSCs in the diseased eye. Furthermore, we reveal the possible cellular mechanisms and identify the associated signaling pathways of MSCs in reversing the pathological conditions of various ocular disorders such as age-related macular degeneration (AMD), retinitis pigmentosa, diabetic retinopathy, and glaucoma. Current stem cell treatment can be dispensed as an independent cell treatment format or with the combination of other approaches. Hence, the improvement of the treatment strategy is largely subjected by our understanding of MSCs mechanism of action.

## 1. Introduction

**Figure 1:**
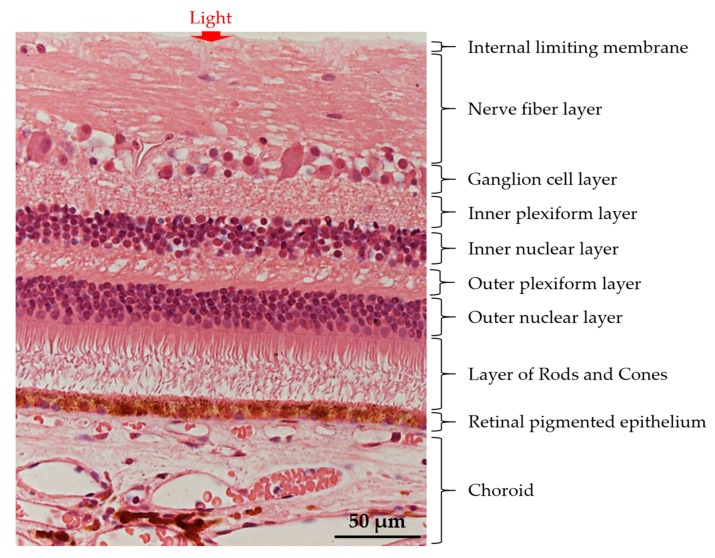
The basic retinal structure. Histological appearance of choroid and retinal layers. The retina is arranged in different layers of cells, from Retinal Pigment Epithelium (RPE), Outer Nuclear Layer (ONL), Outer Plexiform Layer (OPL), Inner Nuclear Layer (INL), Inner Plexiform Layer (IPL), and ganglion cell layer. The retinal layer harbors five retinal neuronal cells, primarily, the rod- and cone-photoreceptors, the Müller glia, the horizontal cell, the bipolar cell, the amacrine cell, and the Retinal Ganglion Cell (RGC). The arrow indicates the light transmission into the retina. Modified with permission from InTech’s Publishing Ethics and Legal Affairs Department [5] (© 2012 Triviño A, De Hoz R, Rojas B, Gallego BI, Ramírez AI, Salazar JJ, Ramírez JM. Published in [short citation] under CC BY 3.0 license. Available from: http://dx.doi.org/10.5772/48359).

In the human eye, visual transmission begins when the light entered is being refracted to the posterior lining of the eye, referred to as the retina [1]. The retina is a conserved structure made up of five distinctive cellular layers of cell bodies and neuropils, comprising of photoreceptors, bipolar, horizontal, amacrine, and ganglion cells; and a supporting glial cell known as Müller glia (Figure 1) [1]. The light signal is first captured by the photoreceptors, which is then distributed along the Outer Nuclear Layer (ONL) of the retina [1]. The photoreceptors share a distinct structure consisting of an array of light-sensing rod and cone photoreceptor cell types, in which they are distinguishable by the light-sensitive, photo-pigment rhodopsin, and opsin, respectively [2]. These membranous photo-pigment proteins are tightly organized in a disc-like membrane to carry out signal transduction in the form of action potential [2]. Individually, the photoreceptors respond to light photon at a specific range of wavelength to achieve hyperpolarization state in the photoreceptor cell’s membrane potential [3]. The photoreceptors convert light signal into electrical impulses and relay these impulses to bipolar cells [4]. The intensity of the relayed impulses were regulated by horizontal cells located in the outer plexiform layer [3]. The synaptic inputs were further relayed to ganglion cells and through the optic nerve into the visual cortex of the brain [4]. This process is known as photo-transduction in which failure will result in visual impairment.

## 2. Current Therapeutic Approach for Retinal Diseases and Its Limitations

Ocular disorder is a universal health condition affecting either the anterior or posterior lining of the eye [6]. Over the years, expanding efforts have been carried out globally by the World Health Organization (WHO) to minimize visual impairment or blindness [6]. Treatment to reduce pathological condition affecting the posterior eye (majority in the retina) deserves greater attention due to the limited accessibility to treatment [6,7].

Retinal degenerative diseases are a group of heterogeneous conditions which include Age-related Macular Degeneration (AMD), retinitis pigmentosa, and diabetic retinopathy [8,9,10,11]. Numerous factors, such as oxidative stress, genetic diseases, light-induced damage, chemical insults, vascular defects or aging, have been suggested to contribute to the development of retinal degeneration [10,12,13,14]. Progressive degeneration of the retinal neurons, predominantly in the photoreceptors, Retinal Ganglion Cells (RGCs), as well as in the Retinal Pigment Epithelium (RPE), could result in severe deterioration of visual function and in due course, permanent visual loss [15,16]. As the mammalian retina has limited self-regenerative nature, visual impairment due to retinal degeneration is difficult to treat [17].

To date, therapeutic options such as surgical and pharmacological interventions are more suitable for patients with early diagnosis to minimize or reduce existing pathological retinal degenerative conditions from further deterioration [18,19]. In addition, some visual prostheses, such as Argus II, which is a cell-free retinal implant that acts on the RGCs to stimulate visual transmission in patients with retinitis pigmentosa or AMD, is costly and users reported difficulties in visual output interpretation [20,21]. In the meantime, results from clinical trials using Food and Drug Administration (FDA) approved anti-Vascular Endothelial Growth Factor (VEGF) drugs, such as Bevacizumab (Avastin) and Ranibizumab (Lucentis), have been reported as promising means to improve visual acuity and maintain retinal anatomy in patients associated with intraocular microvascular complications, such as diabetic retinopathy, retinal vein occlusion, and AMD [22,23]. Anti-VEGF antibodies could selectively bind to VEGF receptors present on the vascularized intraocular tissues—such as the conjunctiva, iris, retina, and RPE—and will therefore prevent massive release of pro-inflammatory cytokines responsible for pathologic intraocular neovascularization [24,25]. Nonetheless, these therapeutic approaches require multiple dosing regimens and repeated delivery via intravitreal injection in order to sustain visual acuity [26]. Wells et al., 2016 also evidenced patients were more prone to Anti-Platelet Trialists’ Collaboration (APTC) events such as nonfatal strokes and vascular deaths following treatment with anti-VEGF antibodies [23]. In the course of exploring possible therapeutic alternatives, increasing evidence of research with Mesenchymal Stem Cells (MSCs), a type of adult stem cells, may prove to be a promising candidate for cell replacement therapy for retinal degenerative diseases.

## 3. Alternative Therapeutic Strategies for Retinal Repair Using Stem Cell-Based Approach

Much effort has been applied to the development of cell replacement therapies to functionally restore and replace lost or damaged tissues or organs that lack intrinsic tissue regenerative responses [27]. Stem cells are a type of cell with high self-renewability and differentiation capability, which are mostly favored to be used as a candidate for cell replacement therapy [28]. In retinal degenerative diseases, research works have been focused on improving the cell recovery and regeneration of terminally-differentiated retinal neuronal cells through delivery of unmodified or modified stem cells by genetic, chemical, or mechanical manipulation [29,30,31,32].

Embryonic Stem Cells (ESCs) hold an astonishing multi-germ layer differentiation potential that can be directed to form almost any cell type of the body [33]. Several clinical trials have advanced to evaluate the efficiency and safety of RPE-derived human ESCs (hESCs) on patients suffering from AMD (NCT01674829) or Stargardt’s Macular Dystrophy (NCT01345006) [34]. The results of the first clinical study was not reported, however, the latter indicated that the patients benefitted from the transplantation and acquired general and peripheral visions by 8–20 points [34]. The investigators further suggested that RPE-derived hESCs could be a potential therapeutic cells that posed no evidence of unfavorable proliferation, immune-rejection, or uneventful systemic and ocular pathological conditions for a period of 22 months following to subretinal transplantation [34]. In another recent study, preliminary data on Phase I/II trial (NCT01344993) reported that patients affected by AMD demonstrated improvement in visual acuity after a year following allogeneic transplantation of pigmented epithelial cells derived from hESCs without any evidence of adverse effect or tumor formation related to the transplanted cells [34]. The results demonstrated that 13 out of the 18 patients showed reconstitution in the RPE structure and improvement in the functional activity [35]. The promising results have led to registration of more new and similar clinical investigations (NCT03046407, NCT02286089, and NCT02755428) to test the efficiency and safety of using RPE-derived hESCs for treatment of AMD. Despite this, their use often evokes ethical issues and requires combination treatment with immunosuppressive drugs to avoid immune rejection towards allogeneic transplant [29,36].

Alternatively, reprogramming somatic cells to a pluripotent state, termed as induced Pluripotent Stem Cells (iPSCs), has ultimately circumvented the risk to graft rejection and avoided ethical controversies in the use of human embryo [37]. The discovery in iPSC technology has regained embryonic-like property in a wide range of human somatic cells with Yamanaka pluripotent transcription factors, consisting of Octamer-binding protein 3/4 (Oct3/4), SRY-box (SOX2), Krüppel-like factor 4 (Klf4), and c-Myc [38]. Several studies have later revealed that cell reprogramming are independent of oncogenic factors, such as c-Myc and Klf4 [39,40], and using only human iPSCs transplanted into patients with exudative macular degeneration showed encouraging outcome in which patients who received iPSC-derived RPE sheet were hindered from further deterioration in visual acuity. However, in 2015, a disconcerting finding of gene mutation in the re-programmed cells prior to the transplantation has led to the termination of subsequent works in the study [39]. It is well documented that the tumorigenic potential in iPSCs, similar to those in ESCs, is the major clinical hurdle for cell-therapies. The risk of cell de-differentiation, genomic instability, and the presence of undifferentiated somatic cells in reprogrammed cells are highly susceptible to malignant transformation [41].

Epigenetic alteration in autologous iPSC-derived cells is capable of provoking T cell or Natural Killer (NK) cell dependent immune rejection prior to teratoma formation [42]. It was suggested that a minute shift in gene expression of transplanted iPSCs will be recognized as foreign antigen by the host defense mechanism to arrest possible tumor development [42,43]. In addition, Kawamura et al., 2016 further implied that the absence of host’s immune surveillance predisposed to the risk of tumorigenicity, whereby iPSC-derived cells were witnessed to form tumor in immunosuppressed allogeneic transplantation model [42]. These results were comparable to what Hsieh et al., 2016 reported in iPSC-conditioned medium [44]. It was also evident that tumor growth may be driven by pluripotent-associated genes [45]. Currently, extensive efforts are being directed to reducing the chance of tumor formation in transplanted ESC and iPSC grafts [46]. As for the seemingly favorable clinical studies, the immunogenicity in iPSCs and the unpredictable risks in the late-onset oncogenic tendencies of the transplanted cells in humans have yet to be ascertained [35,47].

In order to overcome the risk of stem cell rejection during allogeneic or autologous transplantation, the quest continues to focus into stem cells isolated from multipotent, adult stromal cells, referred as MSCs. For cells to be considered as MSCs, they phenotypically express a distinct set of cell surface markers for CD105, CD90, and CD73 but lack CD79α, CD45, CD34, CD19, CD14, CD11b, and Human Leukocyte Antigen class II (HLA-II) [48]. In addition, these cells are able to undergo in vitro tri-lineage differentiation into osteogenic, adipogenic, and chondrogenic, as defined by the International Society for Cellular Therapies (ISCT) guideline for MSCs [48]. Owing to the lack of ethical concerns related to its use, MSCs can be found abundantly in the adult tissues, such as bone marrow, adipose tissue, and dental pulp, as well as in the fetal tissues and fluids, including the umbilical cord-tissue, -blood, and -amniotic fluid [49,50,51,52,53,54]. In addition to their wide distribution, MSCs are also known to possess minimal susceptibility to malignant transformation and are capable of avoiding immune cell recognition, hence providing a potential platform for allogeneic and autologous cell transplants [55].

**Figure 2:**
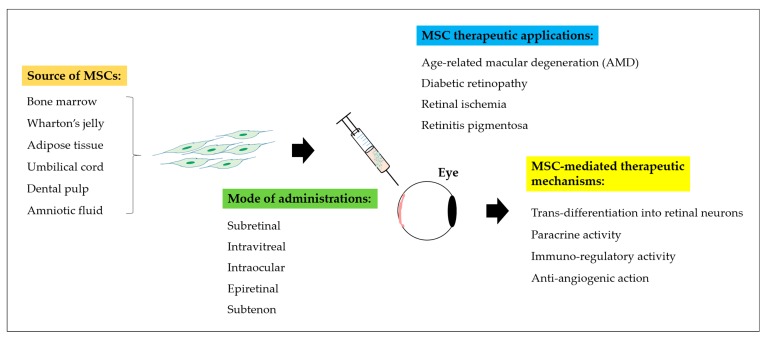
A schematic representation of Mesenchymal Stem Cells (MSCs) therapeutic strategies in retinal degenerative diseases. Different sources of MSC such as bone marrow, Wharton’s jelly, adipose tissue, umbilical cord, dental pulp, and amniotic fluid have been discovered. Multiple routes of administration including subretinal, intravitreal, intraocular, epiretinal or subtenon injections can be implemented to deliver MSCs into the posterior lining of the eye. Delivery of MSCs into patients affected with posterior eye diseases including Age-related Macular Degeneration (AMD), diabetic retinopathy, retinal ischemia, and retinitis pigmentosa can be restored through trans-differentiation, paracrine activity, immuno-regulatory function, and anti-angiogenic action of MSCs.

**Figure 3:**
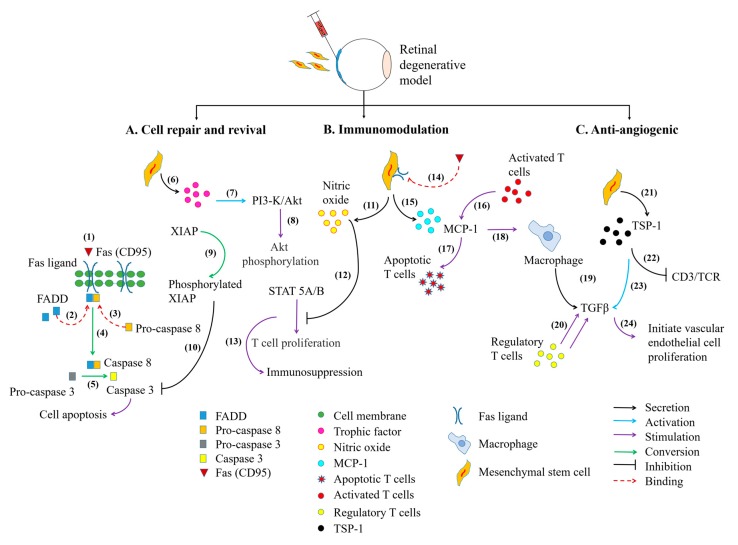
The signaling pathways involved in MSC-mediated therapeutic strategies in the eye. The cell death machinery involves (**1**–**2**) the binding of Fas/Fas ligand, which assembles Fas-Associated protein with Death Domain (FADD) to form a docking site for pro-caspase 8. This event initiates the (**3**–**4**) crosslinking of pro-caspase 8 to FADD and activates caspase 8. Activated caspase 8 (**5**) induces the conversion of pro-caspase 3 into caspase 3 which are essential for the initiation of cell apoptosis. The MSCs cellular reparative action can be exerted (**6**–**7**) by the release of its beneficial trophic factors, including IL-6 which could further promote the migration of MSCs towards site of injury. The binding of IL-6 on MSCs will activate Phosphatidylinositol-3-Kinase (PI3-K)/Akt signaling pathway. (**8**–**10**) The phosphorylated Akt then induces X-linked Inhibitor of Apoptosis Protein (XIAP) phosphorylation leading to inhibition of caspase 3 activity. The immunomodulatory action of MSCs can be depicted through (**11**–**13**) MSCs secretion of nitric oxide, which hampers Signal Transducer Activator-of-Transcription 5 (STAT5) phosphorylation and progressively leads to attenuation of T cell proliferation. The alleviation of T cell activity can be modulated through (**14**–**15**) the expression of Fas ligand on MSC cell surface. This creates binding site for Fas protein, in which induces MSCs secretion of Monocyte Chemotactic Protein-1 (MCP-1) protein. (**16**–**17**) The secreted proteins subsequently attract and induced apoptosis of activated T cells. (**18**–**20**) Accumulation of apoptotic T cells further stimulate macrophage to release Transforming Growth Factor-beta (TGF-β) and subsequently recruit regulatory T cells. The regulatory T cells could also convert cytotoxic T cells into regulatory T cells. In addition, (**21**–**22**) MSCs also secrete Thrombospondin type-1 (TSP-1) proteins to suppress Cluster of Differentiation 3 (CD3)/T cell receptor-mediated T cell proliferation. (**23**–**24**) The released TSP-1 proteins activate TGF-β activity to initiate vascular endothelial cell remodeling. MSC cell differentiation is not mentioned in this figure.

Collectively, MSCs have been widely employed in various acute and chronic neurodegenerative conditions, including central-peripheral neuropathy, stroke, spinal cord injury, as well as ocular degenerative disorders [56,57,58,59]. From the accumulative pre-clinical studies (Table 1) and clinical trials (Table 2), administration of MSCs have revealed significant restoration of the visual system (Figure 2) through MSC-mediated therapeutic mechanisms involving (i) cell differentiation and trans-differentiation processes to replace loss or damaged cells, (ii) paracrine action for cell repair and revival, (iii) modulation of host’s immune responses at inflamed site, and (iv) anti-angiogenic trophic action in certain ocular disorders (Figure 3) [48,54,60].

## 4. MSCs and Its Differentiation for the Treatment of Retinal Diseases

**Figure 4:**
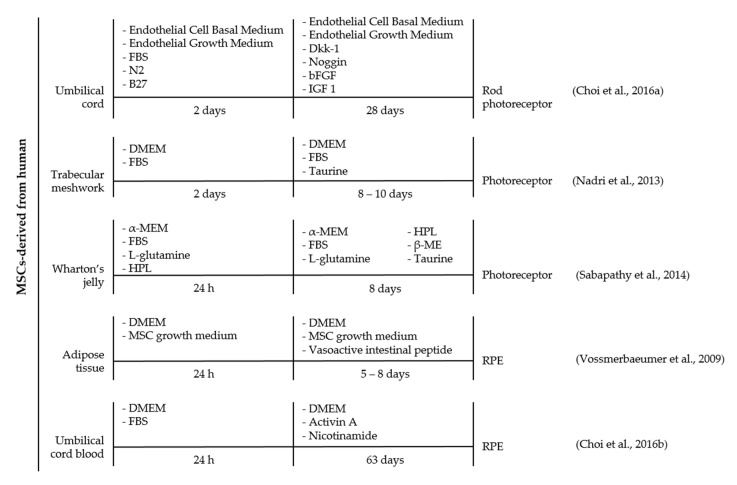
A timeline representation of strategies used to direct human MSCs differentiation into retinal neurons and retinal pigmented epithelial cells, in vitro. Generation of retinal cell from MSCs involve the manipulation of stem cell fate by cytokines, growth factors, or inhibitory peptides. MSCs derived from different tissue origins including bone marrow, dental pulp, umbilical cord, trabecular meshwork, Wharton’s jelly, adipose tissue, and umbilical cord blood have previously demonstrated successful differentiation potential into retinal cells. The vast differentiation potential of MSCs into retinal cells includes photoreceptor, amacrine cell, RGC, and RPE-like cells that requires addition of specific cytokines, growth factors or inhibitory peptides. The differentiation factors are comprised of either taurine, activin A, basic Fibroblast Growth Factor (bFGF), β-mercaptoethanol (β-ME), Dickkopf Wnt signaling pathway inhibitor-1 (Dkk-1), noggin, insulin growth factor 1 (IGF 1), Human Platelet Lysate (HPL), and Vasoactive Intestinal Peptide. FBS, Fetal Bovine Serum; DMEM, Dulbecco’s Modified Eagle Medium; α-MEM, alpha Minimal Essential Medium; B27, B27 supplement; and N2, N2 supplement.

Like all mammals, human retina habitually lacks the ability to regenerate cells [27]. Controlled expression of certain intrinsic and/or extrinsic components regulates mammalian cells from regeneration at certain developmental stages [69]. Opposing this theory, numerous investigations have reported on the regenerative potential of MSC into endodermal and ectodermal lineages, in both in vitro and in vivo models (Figure 4) [70,71]. This lineage-switching phenomenon is referred to as either dedifferentiation or trans-differentiation processes [61]. De-differentiation is an innate regenerative activity, involving the reversion of a terminally differentiated cell into undifferentiated progenitor cell of the same lineage [69]. Meanwhile, trans-differentiation is a two-step differentiation process that involves the dedifferentiation of terminally-differentiated cells and subsequent differentiation into specialized cells of a different lineage [69].

A schematic diagram illustrated various protocols explored on MSCs of different origins to direct cell differentiation into retinal-like lineages, including photoreceptors and RPE cells (Figure 4). It was observed that MSCs can be induced to differentiate independently with taurine [72] or VIP [73] days or through a combination of selective induction cocktails [71,74,75] comprised of Dkk-1, Noggin, bFGF, and IGF 1 [71]; taurine, HPL, and β-ME [74]; or nicotinamide and activin A [71,75]. Meanwhile, using a different protocol on MSCs of similar source were found to develop into retinal neurons or RPE as early as 8 days [73] up to 63 days [75]. The discrepancy in the timeline encourages future optimization to achieve an immediate differentiation capacity at a minimal cost. It is also worth noting that the discrepancy between different protocols may also be influenced by the source [76,77] and isolation method of MSC [78], ontogenetic age [79,80], and others. Previous study observed MSCs derived from the umbilical cord expressed markers for pluripotent stem cells such as Octamer-binding protein 4 (Oct4), ATP-Binding Cassette sub-family G member 2 (ABCG2), Nanog homeobox (Nanog), and SOX2 that are crucial for neurogenic differentiation [78]. On the contrary, a study by Hu et al. evinced a distinct difference in neurogenic potential between human MSCs derived from adipose tissue and umbilical cord [81]. The author showed that cells derived from adipose tissue demonstrated greater efficiency into neuron-like cells up to 45% under retinoic acid induction medium [81]. The inconsistency data suggested the need to further examine the differentiation potential of MSCs from different sources to enable precise selection of MSCs for future regenerative study.

There has also been a debate raised over the poor lineage-switching potential in MSCs and only a marginal population of MSCs was observed to successfully differentiate into the desired cell [82]. One possible explanation has been suggested on the presence of “rare cell” that occupies the residential MSCs niche, termed as multilineage-differentiating stress-enduring (MUSE) cells, in which they represent the subpopulation of successfully differentiated MSCs [82,83]. MUSE cells are pluripotent somatic stem cells that are found intimately tied to the fibroblasts or MSCs of all tissues or organs, while they intrinsically displayed phenotypic markers of ESCs, iPSCs, and MSCs, such as Stage-Specific Embryonic Antigen-3 (SSEA-3), Tumor Resistance Antigen 1-60 (TRA-1-60), Nanog, Oct3/4, and SOX2 [83,84]. These cells displayed a comparable efficiency as ESCs in cell differentiation into multigerm lineages of mesodermal, endodermal, and ectodermal, and devoid of propensity into teratoma formation [83]. Since, MUSE cells are generally present in scarce number, selective isolation and expansion of pure MUSE cell population would elevate the chances to direct differentiation into retinal neurons or RPE and further enhance the efficiency of stem cell-based therapy in various human ocular degenerative disorders.

## 5. Paracrine Activity of MSCs Aids Retinal Cell Repair and Revival

One representative example of MSC paracrine support was provided by a recent study, whereby MSC homing response contributed to visual improvement with profound photoreceptor cells survivability in degenerating Royal College of Surgeons (RCS) rat retina [85]. The study outcome was attributed by trophic peptides released from MSCs to stimulate phagocytic activity of RPE on lethal accumulation of photoreceptor remnants [85]. Besides that, MSC transplantation was found to reduce substantial damage in the posterior lining of the eye and promote cell regeneration through paracrine release of Hypoxia-Inducible Factor-1α (HIF-1α) and axonal Growth-Associated Protein-43 (GAP–43), respectively [49].

The therapeutic effect of MSCs was also identified from the neurotrophic action of Ciliary Neurotrophic Factor (CNTF) and Brain-Derived Neurotrophic Factor (BDNF) secreted from MSCs in an oxidative-induced RGCs culture, and have led to the downregulation of pro-inflammatory cytokines, such as Tumor Necrosis Factor-α (TNF-α) and Interleukin-1β (IL-1β) release [86]. Mead et al., 2014 previously evidenced that MSCs exerted protective action in axotomized rat RGCs and promoted neurite formation, through paracrine release of Prostaglandin E2 Receptor (PGE2R) and IL-6-mediated growth factors, such as Platelet-Derived Growth Factor (PDGF) and Nerve Growth Factor (NGF) [87]. A similar trial was also reported that neurotrophic factors, such as NGF, bFGF, and Glial Cell Line-Derived Neurotrophic Factor (GDNF), released from adipose tissue-derived MSCs were found to preserve retinal ganglion cell survivability and reduced oxidative stress damage in the retina [51]. Furthermore, integrated MSCs were detected to differentiate into retinal astrocytes, RGCs, and pericytes in a diabetic-induced mice model [51]. The study outcome was further postulated in an induced diabetic retinopathy rat model, whereby successfully engrafted MSCs demonstrated selective protection against retinal gliosis along with the restoration of retinal vascular integrity and functionality and simultaneously differentiated into Müller glia [62]. These pre-clinical studies have confidently shown the promising use of MSCs in retinal degenerative disorders, most importantly, administration of MSCs were also found to illustrate infinite differentiation potential beyond its mesodermal origin.

Apart from the secretory cytokines and growth factors, there has been increasing evidence reported on the therapeutic potential of extracellular vesicles released from the MSCs [88,89]. Based on their distinct biological composition, extracellular vesicles can be classified into microvesicles, microparticles or exosomes, in which they play a crucial role in modulating cell-to-cell communication in a paracrine manner [88,90]. Considerable attention has been focused on MSC-derived exosomes, as these molecules are well-enriched with enzymes, which carry out protective responses in ocular degenerative disorders [88]. It was previously identified that the intravitreal delivery of exosomes-derived from MSCs exhibited restorative and protective actions in mouse models of retinal laser injury [91]. The author reported that transplanted exosomes were found to inhibit inflammatory-mediated cytokines infiltration, including Monocyte Chemotactic Protein-1 (MCP-1), TNF-α, and Intercellular Adhesion Molecule-1 (ICAM-1), hence, dampened macrophage- and T cell-mediated immune responses [91].

## 6. MSCs Alleviates Inflammation in Retinal Diseases

The human eye is a sophisticated organ that holds innate property in modulating immune responses in the ocular microenvironment. It has been described that parenchymal cells in the eye—particularly from the iris, ciliary body, and RPE cells—are able to suppress the intraocular immune reactivity, which makes the eye an immune-privileged site [92,93]. This innate feature in the eye is associated with the presence of immune modulators such as Fas ligand (CD95), Programmed Death Ligand (PDL)-1, Cytotoxic T-Lymphocyte Antigen 2 (CTLA-2), and CTLA-4 [93]. In addition, the presence of blood-aqueous barrier and Blood-Retina Barrier (BRB) that lack lymphatic drainage system could further limit possible migration of immune cells across the eye [93].

Dysregulation of the intraocular immune system is a pathological condition commonly manifested in AMD, glaucoma, diabetic retinopathy, and uveitis [94]. It is represented by a profound release of pro-inflammatory cytokines, chemokines, Matrix Metalloproteinases (MMPs), that progressively results in the loss of endothelium tight junction proteins, destruction, and leakage of BRB, hence, facilitating the infiltration of immune cells [95]. It is also believed that the privileged status of the eye may be compromised when predisposed to autoimmune reaction against self-antigens, for instance, uveal melanin, arrestin, interphotoreceptor retinoid-binding protein (IRBP), and recoverin, which are expressed in the retina, lens, and cornea [93,96]. This, in turn, will trigger subset of antigen-activated Cluster of Differentiation 4 (CD4) T cells to release transcription factors that are essential for downstream activation of autoimmune-associated T helper cell proliferation, such as IL-12 and Interferon-γ (IFN-γ) for T helper type 1 (T_h_1) cells and IL-6, IL-21, IL-23, and Transforming Growth Factor-β (TGF-β) for T_h_17 cells [96].

Sufficient number of studies have shown the capability of MSCs as a potent immunotherapeutic candidate to modulate both the innate and adaptive immune responses and suppress immunoreactivity in a broad range of diseases, including the eye [16,53]. Their immune evasive status could be attributed to the absence of activated T cells-associated ligands for HLA-I and co-stimulatory molecules from HLA-II, including CD40, CD80, and CD86, which are responsible in triggering graft rejection through T cell activation [55]. Also, these cells are capable to downregulate allogeneic lymphocyte proliferation and stimulate T regulatory cells expression [55,97]. Henceforth, MSCs are likely to obviate the major hurdles seen in cell therapy with ESCs and iPSCs [59,98]. In addition to their great potential, MSCs also have the advantages of fewer ethical concerns and less immune rejection. For example, Lee et al., 2015 has recently illustrated a significant preservation of the local niche of an inflammation-mediated murine eye model after periorbital delivery of MSCs [99]. The study showed that the restorative effect was accompanied by downregulation of pro-inflammatory cytokines activities and CD4 T cells infiltration [99]. Meanwhile, comparable study reported that MSCs were able to promote suppressive role of regulatory T cells and Forkhead Box P3 (FOXP3) transcription factor on T cell response via paracrine release of TGF-β from MSCs [100,101,102].

It has been reviewed that degeneration of RGC axons is a secondary phenomenon that occurs in patients with glaucoma that can be caused by retinal neovascularization or excessive accumulation of glutamate in the retina [103,104]. Based on an experimental study on glaucomatous rat model, the author reported an improvement in RGCs survivability following to intravitreal MSC transplantation [105]. The successfully integrated MSCs were found to reside into the ganglion cell layer and the INL, in which they influenced the host’s immune response by suppressing pro-inflammatory cytokines production for interferon-γ and TNF-α via activation of IL-1 Receptor Antagonist (IL-1RA) and PGE2R [85].

## 7. MSCs Modulates Angiogenic Activity in Retinal Diseases

Sprouting of new blood vessels from pre-existing vessels through the angiogenesis process has been recognized in several types of ocular disorders, including diabetic retinopathy, retinopathy of prematurity, retinal vein occlusion, AMD, and glaucoma [8,16,54,106]. The occurrence of intraocular angiogenesis is further orchestrated by the increase of proangiogenic factors, mainly VEGF in the retinal neurons and RPE cells [95]. Untreated condition in the patients can lead to massive degradation in the basal endothelium membrane, neovascularization, and functional disruption of the neurosensors in the retina [107].

Among the vast potential of MSCs, substantial pre-clinical and clinical studies have equally validated on the therapeutic potential of MSCs in restoration of ocular neovascularization. In a recent study, subconjunctival injection bone marrow-derived MSCs into chemically-induced rat cornea where was found to encourage corneal wound healing and stabilized neovascularization lesion through suppression of VEGF, Matrix Metalloproteinase 9 (MMP-9), and Toll-Like Receptors (TLRs), which presumably contributes to the downregulation of pro-inflammatory cytokine production [108]. In an oxygen-induced retinopathy mouse model, intraperitoneal transplantation of human placental amniotic membrane-derived MSCs efficiently homed to and engrafted onto the injured site with profound release of angiogenic factor, Transforming Growth Factor-β1 (TGF-β1) from MSCs [54]. Astonishingly, upregulation of TGF-β1 level significantly decreased endothelial cell proliferation devoid of neovascularization while restoring retinal angiogenesis [54].

It is noteworthy that metabolic dysregulation in diabetic patients involves an uncontrollable increase in blood glucose (hyperglycemia) and cholesterol (hyperlipidemia) levels which stimulate VEGF secretion and ultimately contribute to the pathological alteration in the retinal vasculature integrity [107]. Ezquer et al., 2016 previously reported no apparent changes in pro-angiogenic expression observed upon administration of MSC in diabetic-induced mice model [51]. In this study, the author reported that MSCs exerted cytoprotective action through secretion of platelet-derived anti-angiogenic factor, Thrombospondin Type-1 (TSP-1), which is also produced primarily from the ocular surface epithelium, including the RPE, choroid, and Müller glial cells [51,109,110,111]. TSP-1 is a glycoprotein that has been found to modulate MSC functions including anti-angiogenic, anti-inflammatory, as well as immunomodulatory and immune-privileged activities in a healthy ocular microenvironment [51]. This data was further supported by a study conducted on an induced diabetic retinopathy rat model, whereby administration of bone marrow derived-MSCs demonstrated selective protection against retinal gliosis, increased vascular integrity, and retinal function [62]. At the same time, treated diabetic eyes demonstrated selective MSCs integration and differentiation into Müller glia, in comparison to the healthy sham eyes [62]. In another study conducted on diabetic-induced mice model, there was no sign of neovascularization, destruction of retinal vasculature, RGC loss or increase in pro-angiogenic factors upon intravitreal administration of adipose-derived MSCs [51].

In a preceding study [106], MSCs secreted a wide range of growth factors and cytokines as well as other proteolytic and angiogenic proteins—including VEGF, bFGF, TGF-β1, Stromal Cell-Derived Factor 1 (SDF-1), cathepsin, MMPs, and Plasminogen Activator Inhibitor 1 (PAI-1)—in response to tissue repair. The expression of these secretory proteins from MSCs were highly correlated to the onset of pathological neovascularization in the eye. A study by Hou et al., 2010 previously demonstrated that bone marrow-derived MSCs selectively migrated and engrafted into choroidal neovascularization lesions and further exaggerated the pathological condition [112]. A similar response has been reported on the secretory profile of diabetic rat bone marrow-derived MSCs were capable to induce angiogenic potential in in vitro endothelial cells culture via upregulation of secretion of local angiogenic mediators, such as Insulin-Like Growth Factor 1 (IGF-1) and Latent Transforming Growth Factor B Binding Protein type 1 (LTβP-1) [113]. Nevertheless, it was reported that subsequent expression of VEGF was found ameliorated, with an undetectable level of inflammatory proteins, such as TNF-α and FGF [113]. The suppression of inflammatory response could be contributed to the release of anti-angiogenic factors, such as TSP-1, from MSCs [51]. Chu et al., 2013 also observed similar suppression in VEGF activity, which was attributed to indirect inhibition of TSP-1 on VEGF receptor, via binding to CD36 and subsequent recruitment of SHP-1 onto VEGF target [114].

## 8. Conclusions

The limitation of self-reparative and regenerative capacity of retinal cells has opened up a new avenue for mesenchymal stem cell-based therapy for the treatment of ocular disorders including age-related macular degeneration, diabetic retinopathy, retinopathy of prematurity, and glaucoma. Mesenchymal stem cells have emerged as a valuable tool in cell replacement therapy due to the lack of ethical issues, easy isolation, and expansion and its privilege to escape from immune cell surveillance. MSCs have shown promising outcomes in cell regeneration through several mechanisms involving differentiation competency to replace loss or injured cells either by in vitro culture with selective growth factor or by the influence of local microenvironment regulatory inputs. We have also recapitulated the influence of paracrine network in MSCs that govern its reparative response through the release of restorative trophic factors and cytokines and further engaged on the modulatory signaling action of MSC in immune cells, specifically on T cell response. Lastly, the trophic factors or cytokines could also exert anti-angiogenic property involved in the restoration of ocular vasculopathies.

## Figures and Tables

**Table 1:** Recent pre-clinical studies MSCs for the treatment of retinal diseases.

Disease Target	Source of MSCs	Experimental Design (Route of Delivery; Cell Concentration)	Research Outcomes	References
AMD	Rat bone marrow	Subretinal; 1.0 × 10^6^ cells/eye	Integrated MSCs enhanced retinal cell survivability, architecture, and functionality of induced retinal degeneration rat model through MSC differentiation and replacement of loss RPE.	[61]
Diabetic retinopathy	Rat bone marrow	Intravitreal; 1.0 × 10^5^ cells/µL	MSCs were found to integrate mostly in the diabetic eyes with reduction in retinal gliosis followed by an increased in retinal function.	[62]
	Mouse adipose tissue	Intravitreal; 1.0 × 10^5^ cells/µL	MSCs regenerated into retinal astrocytes and RGC, and protected RGC from oxidative damage through secretion of MSC factors comprising of NGF, bFGF, and GDNF.	[51]
Retinal ischemia	Human bone marrow	Intravitreal; Not available	Administration of hypoxic-conditioned medium from MSCs in rat model of retinal ischemia promoted RGC survivability and restored retinal function through MSC paracrine effect.	[50]
	Not available	Intraocular; 1.0 × 10^4^ cells/µL	Engrafted MSCs improved RGC survivability after retinal ischemic injury in a mouse model.	[63]
Retinal degeneration	Human bone marrow	Subretinal and intravitreal; 5.0 × 10^5^ cells/µL	MSCs increased photoreceptor cell survivability from degeneration and sustained retinal function in retinal degenerating rat model.	[64]
		Epiretinally; 5 × 10^4^ cells/µL		[65]
		Subretinal; 2.5 × 10^4^ cells/µL		[66]
	Human umbilical cord blood	Subretinal; Not available	A significant preservation of degenerating rat retinal photoreceptors, function, and architecture through secretion of MSC neurotrophic factors, such as IL-6, FGF2, and BDNF.	[67]
Glaucoma	Rat bone marrow	In vitro co-culture system; Not available	In vitro co-cultured of MSCs with hypoxic-induced rat RGC exerted anti-apoptotic effect on RGC via reduction in caspase-3 activity.	[68]
	Human dental pulp, bone marrow, and adipose tissue	Intravitreal; 3.0 × 10^4^ cells/µL	MSCs derived from human dental pulp and bone marrow increased RGC survivability and restored retinal function in ocular-induced hypertensive rat model.	[53]

MSCs, Mesenchymal Stem Cells; RPE, Retinal Pigment Epithelium; RGC, Retinal Ganglion Cell; NGF, Nerve Growth Factor; bFGF, basic Fibroblast Growth Factor; GDNF, Glial Cell Line-Derived Neurotrophic Factor; IL-6, Interleukin-6; FGF2, Fibroblast Growth Factor 2; BDNF, Brain-Derived Neurotrophic Factor.

**Table 2:** Current clinical trials using MSCs for the treatment of retinal diseases.

Application	Source of MSCs	Experimental Design		Clinical Phases	Clinical Trials Identifier (ClinicalTrials.gov)
		Route of Delivery	Concentration of Stem cells		
i. AMD	Umbilical tissue	Subretinal	6.0 × 10^4^ cells–3.0 × 10^5^ cells	Phase 1/2a	NCT01226628
	Bone marrow	Intravitreal	Not available	Phase 1/2	NCT02016508
	Bone marrow	Intravitreal	3.4 × 10^6^ cells/0.1 mL	Phase 1	NCT01736059
	Bone marrow	Intravitreal	10.0 × 10^6^ cells/0.1 mL	Phase 1/2	NCT01518127
ii. Retinitis pigmentosa	Bone marrow	Intravitreal	3.4 × 10^6^ cells/0.1 mL	Phase 1	NCT01736059
	Bone marrow	Intravitreal	1.0 × 10^6^ cells/0.1 mL	Phase 1	NCT01531348
	Bone marrow	Intravitreal	10.0 × 10^6^ cells/0.1 mL	Phase 2	NCT01560715
	Bone marrow	Intravitreal	3.4 × 10^6^ cells/0.1 mL	Phase 1	NCT01736059
	Bone marrow	Intravitreal	10.0 × 10^6^ cells/0.1 mL	Phase 1	NCT01068561
iii. Diabetic retinopathy	Bone marrow	Intravitreal	3.4 × 10^6^ cells/0.1 mL	Phase 1	NCT01736059
iv. Ischemic retinopathy	Bone marrow	Intravitreal	10.0 × 10^6^ cells/0.1 mL	Phase 1/2	NCT01518842

Phase 2a defines a pilot clinical trial for the evaluation of efficacy (and safety) in selective groups of patient subjects with associated disease or condition to be treated, diagnosed, or prevented.
